# Supplementary material for: Self-Perceived Health among Migrants Seen in Médecins du Monde Free Clinics in Europe: Impact of Length of Stay and Wealth of Country of Origin on Migrants’ Health
Source: Int J Environ Res Public Health. 2019 Dec 4;16(24):4878. doi: 10.3390/ijerph16244878 (PMC6950051; doi:10.3390/ijerph16244878)
Supplement: Supplementary file 1 [file ijerph-16-04878-s001.zip › IJERPH 2019 - 12 - 02/Full Results Interactions Phy S5.odt]

Table S5 Full Results for Physical SPH with and without interaction.

|  | Physical Health |
| --- | --- |
|  | Female | Male |
|  | No interaction | Interaction | No interaction | Interaction |
| Age group |  |  |  |  |
| 1st quartile [18;28[ | Ref. | Ref. | Ref. | Ref. |
| 2nd quartile [28;34[ | 1.05 [0.59; 1.87] | 1.05 [0.58; 1.88] | 0.91 [0.59; 1.40] | 0.91 [0.59; 1.40] |
| 3rd quartile [34;44[ | 0.63 [0.35; 1.15] | 0.64 [0.35; 1.16] | 0.62 [0.40; 0.97]* | 0.62 [0.40; 0.97]* |
| 4th quartile [44;85] | 0.65 [0.35; 1.21] | 0.65 [0.35; 1.21] | 0.63 [0.37; 1.10] | 0.63 [0.37; 1.10] |
| Surveyed Countries |  |  |  |  |
| Germany | Ref. | Ref. | Ref. | Ref. |
| Switzerland | 5.56 [0.73; 42.37] | 6.10 [0.80; 46.32] | 1.15 [0.27; 4.82] | 1.15 [0.27; 4.84] |
| Spain | 0.55 [0.14; 2.12] | 0.60 [0.15; 2.31] | 2.18 [0.72; 6.57] | 2.19 [0.72; 6.67] |
| Turkey | 0.91 [0.19; 4.37] | 0.97 [0.20; 4.70] | 1.64 [0.42; 6.50] | 1.65 [0.42; 6.52] |
| United Kingdom | 0.39 [0.09; 1.68] | 0.40 [0.09; 1.75] | 0.63 [0.16; 2.48] | 0.63 [0.16; 2.50] |
| Region of Origin |  |  |  |  |
| Middle East | Ref. | Ref. | Ref. | Ref. |
| Africa | 3.47 [0.87; 13.78] | 3.64 [0.92; 14.36] | 1.37 [0.57; 3.29] | 1.37 [0.57; 3.29] |
| America | 7.65 [1.56; 37.47]* | 8.14 [1.67; 39.64]* | 0.74 [0.22; 2.43] | 0.73 [0.22; 2.43] |
| Asia | 3.06 [0.68; 13.70] | 3.48 [0.78; 15.57] | 1.99 [0.77; 5.12] | 1.99 [0.77; 5.12] |
| Europe | 2.38 [0.45; 12.50] | 2.37 [0.45; 12.41] | 1.55 [0.43; 5.61] | 1.55 [0.43; 5.60] |
| Maghreb | 3.94 [0.73; 21.18] | 4.29 [0.80; 22.88] | 1.73 [0.59; 5.10] | 1.73 [0.59; 5.10] |
| Immigration Status |  |  |  |  |
| Residence permit | Ref. | Ref. | Ref. | Ref. |
| Undocumented | 0.86 [0.55; 1.36] | 0.88 [0.56; 1.39] | 0.89 [0.61; 1.31] | 0.89 [0.61; 1.31] |
| Asylum Seeker | 0.68 [0.42; 1.10] | 0.69 [0.43; 1.13] | 0.56 [0.36; 0.86]* | 0.56 [0.36; 0.86]* |
| Housing conditions |  |  |  |  |
| Instable | Ref. | Ref. | Ref. | Ref. |
| Stable | 1.76 [1.15; 2.69]* | 1.77 [1.16; 2.71]* | 1.27 [0.90; 1.81] | 1.27 [0.90; 1.81] |
| Income satisfaction |  |  |  |  |
| Not Enough | Ref. | Ref. | Ref. | Ref. |
| Enough | 1.36 [0.74; 2.53] | 1.35 [0.73; 2.50] | 0.94 [0.44; 1.98] | 0.94 [0.44; 1.98] |
| Job |  |  |  |  |
| No | Ref. | Ref. | Ref. | Ref. |
| Yes | 1.37 [0.86; 2.17] | 1.39 [0.87; 2.21] | 1.90 [1.30; 2.76]* | 1.90 [1.30; 2.76]* |
| Insurance coverage |  |  |  |  |
| Yes | Ref. | Ref. | Ref. | Ref. |
| No | 1.44 [0.58; 3.59] | 1.42 [0.57; 3.53] | 0.67 [0.29; 1.54] | 0.67 [0.29; 1.54] |
| Length of Stay and Human Development Index |  |  |  |  |
| Human Development Index above 0.5 | 1.94 [1.06; 3.56]* | 5.96 [1.61; 22.06]* | 1.41 [0.93; 2.13] | 1.46 [0.61; 3.49] |
| LOS <3 months | 1.96 [1.05; 3.65]* | 4.72 [1.48; 15.06]* | 0.79 [0.50; 1.25] | 0.81 [0.37; 1.76] |
| Interaction |  | 0.26 [0.07; 1.01] |  | 0.96 [0.37; 2.47] |
| AIC | 669.98 | 667.57 | 937.37 | 939.36 |
| BIC | 761.57 | 763.52 | 1035.05 | 1041.69 |
| Log Likelihood | -313.99 | -311.79 | -447.68 | -447.68 |
| Deviance | 627.98 | 623.57 | 895.37 | 895.36 |
| Num. obs. | 579 | 579 | 774 | 774 |
| * 1 outside the confidence interval |
| Statistical models |
