# Supplementary material for: Self-Perceived Health among Migrants Seen in Médecins du Monde Free Clinics in Europe: Impact of Length of Stay and Wealth of Country of Origin on Migrants’ Health
Source: Int J Environ Res Public Health. 2019 Dec 4;16(24):4878. doi: 10.3390/ijerph16244878 (PMC6950051; doi:10.3390/ijerph16244878)
Supplement: Supplementary file 1 [file ijerph-16-04878-s001.zip › IJERPH 2019 - 12 - 02/Full Results Interactions Psy S6.odt]

Table S6 Full Results for Mental SPH with and without interaction.

|  | Mental Health |
| --- | --- |
|  | Female | Male |
|  | No interaction | Interaction | No interaction | Interaction |
| Age group |  |  |  |  |
| 1st quartile [18;28[ | Ref | Ref | Ref | Ref |
| 2nd quartile [28;34[ | 0.52 [0.27; 1.03] | 0.52 [0.27; 1.03] | 0.89 [0.50; 1.56] | 0.88 [0.50; 1.55] |
| 3rd quartile [34;44[ | 0.63 [0.34; 1.16] | 0.63 [0.34; 1.16] | 1.02 [0.58; 1.77] | 1.01 [0.58; 1.75] |
| 4th quartile [44;85] | 0.59 [0.32; 1.10] | 0.59 [0.31; 1.09] | 0.85 [0.47; 1.54] | 0.84 [0.46; 1.53] |
| Surveyed Countries |  |  |  |  |
| Germany | Ref | Ref | Ref | Ref |
| Switzerland | 4.66 [0.60; 36.39] | 4.60 [0.59; 35.67] | 2.49 [0.54; 11.59] | 2.57 [0.55; 11.99] |
| Spain | 0.77 [0.22; 2.73] | 0.78 [0.22; 2.77] | 2.74 [0.77; 9.67] | 2.89 [0.81; 10.36] |
| Turkey | 0.08 [0.02; 0.39]* | 0.08 [0.02; 0.39]* | 0.10 [0.02; 0.50]* | 0.11 [0.02; 0.51]* |
| United Kingdom | 0.31 [0.08; 1.25] | 0.31 [0.08; 1.24] | 0.89 [0.20; 3.95] | 0.92 [0.21; 4.12] |
| Region of Origin |  |  |  |  |
| Middle East | Ref | Ref | Ref | Ref |
| Africa | 2.29 [0.51; 10.27] | 2.40 [0.53; 10.85] | 0.91 [0.36; 2.30] | 0.88 [0.35; 2.25] |
| America | 2.76 [0.53; 14.31] | 2.92 [0.56; 15.29] | 0.47 [0.14; 1.63] | 0.45 [0.13; 1.58] |
| Asia | 3.98 [0.85; 18.57] | 4.34 [0.91; 20.69] | 1.73 [0.72; 4.18] | 1.72 [0.71; 4.16] |
| Europe | 2.51 [0.49; 12.93] | 2.60 [0.50; 13.50] | 0.91 [0.25; 3.33] | 0.90 [0.25; 3.29] |
| Maghreb | 3.47 [0.61; 19.67] | 3.73 [0.65; 21.45] | 0.99 [0.33; 3.00] | 0.96 [0.32; 2.92] |
| Immigration Status |  |  |  |  |
| Residence permit | Ref | Ref | Ref | Ref |
| Undocumented | 0.78 [0.49; 1.24] | 0.79 [0.49; 1.26] | 0.70 [0.46; 1.09] | 0.71 [0.46; 1.09] |
| Asylum Seeker | 0.75 [0.45; 1.25] | 0.76 [0.45; 1.27] | 0.54 [0.32; 0.91]* | 0.54 [0.32; 0.91]* |
| Housing conditions |  |  |  |  |
| Instable | Ref | Ref | Ref | Ref |
| Stable | 1.98 [1.25; 3.13]* | 1.97 [1.25; 3.11]* | 1.62 [1.06; 2.47]* | 1.61 [1.06; 2.46]* |
| Income satisfaction |  |  |  |  |
| Not Enough | Ref | Ref | Ref | Ref |
| Enough | 1.20 [0.67; 2.17] | 1.20 [0.67; 2.17] | 0.44 [0.20; 0.96]* | 0.44 [0.20; 0.95]* |
| Job |  |  |  |  |
| No | Ref | Ref | Ref | Ref |
| Yes | 1.53 [0.92; 2.54] | 1.53 [0.92; 2.54] | 2.10 [1.30; 3.41]* | 2.12 [1.31; 3.45]* |
| Insurance coverage |  |  |  |  |
| Yes | Ref | Ref | Ref | Ref |
| No | 1.78 [0.75; 4.24] | 1.77 [0.74; 4.23] | 1.63 [0.71; 3.74] | 1.63 [0.71; 3.76] |
| Length of Stay and Human Development Index |  |  |  |  |
| Human Development Index above 0.5 | 0.90 [0.41; 1.97] | 1.29 [0.35; 4.72] | 1.02 [0.55; 1.88] | 1.38 [0.41; 4.64] |
| LOS <3 months | 0.73 [0.39; 1.38] | 1.00 [0.33; 3.00] | 0.75 [0.41; 1.36] | 0.98 [0.32; 2.96] |
| Interaction |  | 0.62 [0.16; 2.35] |  | 0.68 [0.18; 2.54] |
| AIC | 610.54 | 612.02 | 698.89 | 700.55 |
| BIC | 702.09 | 707.93 | 796.52 | 802.83 |
| Log Likelihood | -284.27 | -284.01 | -328.45 | -328.28 |
| Deviance | 568.54 | 568.02 | 656.89 | 656.55 |
| Num. obs. | 578 | 578 | 772 | 772 |
| * 1 outside the confidence interval |
| Statistical models |
