# Supplementary material for: Self-Perceived Health among Migrants Seen in Médecins du Monde Free Clinics in Europe: Impact of Length of Stay and Wealth of Country of Origin on Migrants’ Health
Source: Int J Environ Res Public Health. 2019 Dec 4;16(24):4878. doi: 10.3390/ijerph16244878 (PMC6950051; doi:10.3390/ijerph16244878)
Supplement: Supplementary file 1 [file ijerph-16-04878-s001.zip › IJERPH 2019 - 12 - 02/Result Sensitivity Analysis Phy Interactions S7.odt]

Table S7 Sensitivity analysis for Physical SPH, interactions using different cut-off points for Human Development Index.

|  | Physical Self-Perceived Health |
| --- | --- |
|  | Females | Males |
|  | Model 1 | Model 2 | Model 3 | Model 4 | Model 5 | Model 6 | Model 7 | Model 8 |
|  |  |  |  |  |  |  |  |  |
|  |  |  |  |  |  |  |  |  |
| 2nd quartile [28;34[ | 1.05 | 1.05 | 1.09 | 1.13 | 0.91 | 0.91 | 0.88 | 0.90 |
|  | [0.59; 1.87] | [0.58; 1.88] | [0.61; 1.96] | [0.63; 2.03] | [0.59; 1.40] | [0.59; 1.40] | [0.58; 1.36] | [0.58; 1.37] |
| 3rd quartile [34;44[ | 0.63 | 0.64 | 0.66 | 0.68 | 0.62* | 0.62* | 0.60* | 0.61* |
|  | [0.35; 1.15] | [0.35; 1.16] | [0.36; 1.20] | [0.37; 1.24] | [0.40; 0.97] | [0.40; 0.97] | [0.38; 0.94] | [0.39; 0.95] |
| 4th quartile [44;85] | 0.65 | 0.65 | 0.67 | 0.71 | 0.63 | 0.63 | 0.63 | 0.64 |
|  | [0.35; 1.21] | [0.35; 1.21] | [0.36; 1.27] | [0.38; 1.34] | [0.37; 1.10] | [0.37; 1.10] | [0.36; 1.10] | [0.37; 1.11] |
| Switzerland | 5.56 | 6.10 | 6.36 | 6.83 | 1.15 | 1.15 | 1.02 | 0.92 |
|  | [0.73; 42.37] | [0.80; 46.32] | [0.79; 50.96] | [0.83; 56.21] | [0.27; 4.82] | [0.27; 4.84] | [0.24; 4.30] | [0.21; 3.97] |
| Spain | 0.55 | 0.60 | 0.66 | 0.78 | 2.18 | 2.19 | 1.63 | 1.55 |
|  | [0.14; 2.12] | [0.15; 2.31] | [0.17; 2.60] | [0.19; 3.24] | [0.72; 6.57] | [0.72; 6.67] | [0.52; 5.10] | [0.48; 4.97] |
| Turkey | 0.91 | 0.97 | 0.96 | 1.07 | 1.64 | 1.65 | 1.39 | 1.28 |
|  | [0.19; 4.37] | [0.20; 4.70] | [0.20; 4.71] | [0.21; 5.39] | [0.42; 6.50] | [0.42; 6.52] | [0.35; 5.54] | [0.31; 5.19] |
| United Kingdom | 0.39 | 0.40 | 0.42 | 0.47 | 0.63 | 0.63 | 0.53 | 0.51 |
|  | [0.09; 1.68] | [0.09; 1.75] | [0.10; 1.88] | [0.10; 2.16] | [0.16; 2.48] | [0.16; 2.50] | [0.13; 2.11] | [0.13; 2.05] |
| Africa | 3.47 | 3.64 | 3.52 | 2.81 | 1.37 | 1.37 | 1.26 | 1.26 |
|  | [0.87; 13.78] | [0.92; 14.36] | [0.86; 14.41] | [0.73; 10.87] | [0.57; 3.29] | [0.57; 3.29] | [0.53; 3.00] | [0.53; 2.98] |
| America | 7.65* | 8.14* | 6.09* | 6.10* | 0.74 | 0.73 | 1.08 | 1.13 |
|  | [1.56; 37.47] | [1.67; 39.64] | [1.12; 33.03] | [1.17; 31.81] | [0.22; 2.43] | [0.22; 2.43] | [0.29; 4.02] | [0.31; 4.13] |
| Asia | 3.06 | 3.48 | 3.08 | 3.27 | 1.99 | 1.99 | 2.34 | 2.27 |
|  | [0.68; 13.70] | [0.78; 15.57] | [0.65; 14.70] | [0.74; 14.42] | [0.77; 5.12] | [0.77; 5.12] | [0.88; 6.20] | [0.89; 5.83] |
| Europe | 2.38 | 2.37 | 1.75 | 1.30 | 1.55 | 1.55 | 2.05 | 2.44 |
|  | [0.45; 12.50] | [0.45; 12.41] | [0.30; 10.16] | [0.21; 8.26] | [0.43; 5.61] | [0.43; 5.60] | [0.51; 8.21] | [0.58; 10.23] |
| Maghreb | 3.94 | 4.29 | 3.16 | 3.81 | 1.73 | 1.73 | 2.42 | 2.29 |
|  | [0.73; 21.18] | [0.80; 22.88] | [0.53; 18.79] | [0.71; 20.52] | [0.59; 5.10] | [0.59; 5.10] | [0.73; 8.01] | [0.76; 6.92] |
| Undocumented | 0.86 | 0.88 | 0.86 | 0.92 | 0.89 | 0.89 | 0.90 | 0.90 |
|  | [0.55; 1.36] | [0.56; 1.39] | [0.54; 1.35] | [0.58; 1.45] | [0.61; 1.31] | [0.61; 1.31] | [0.61; 1.32] | [0.61; 1.31] |
| Asylum Seeker | 0.68 | 0.69 | 0.63 | 0.65 | 0.56* | 0.56* | 0.53* | 0.53* |
|  | [0.42; 1.10] | [0.43; 1.13] | [0.39; 1.02] | [0.40; 1.05] | [0.36; 0.86] | [0.36; 0.86] | [0.34; 0.82] | [0.34; 0.81] |
| Stable Accomodation | 1.76* | 1.77* | 1.73* | 1.78* | 1.27 | 1.27 | 1.31 | 1.30 |
|  | [1.15; 2.69] | [1.16; 2.71] | [1.13; 2.65] | [1.16; 2.71] | [0.90; 1.81] | [0.90; 1.81] | [0.92; 1.86] | [0.92; 1.85] |
| Income satisfaction Enough | 1.36 | 1.35 | 1.46 | 1.49 | 0.94 | 0.94 | 0.95 | 0.94 |
|  | [0.74; 2.53] | [0.73; 2.50] | [0.79; 2.72] | [0.80; 2.78] | [0.44; 1.98] | [0.44; 1.98] | [0.45; 2.01] | [0.44; 1.99] |
| Job | 1.37 | 1.39 | 1.30 | 1.34 | 1.90* | 1.90* | 1.86* | 1.86* |
|  | [0.86; 2.17] | [0.87; 2.21] | [0.82; 2.05] | [0.85; 2.13] | [1.30; 2.76] | [1.30; 2.76] | [1.28; 2.71] | [1.28; 2.70] |
| Not Covered | 1.44 | 1.42 | 1.43 | 1.43 | 0.67 | 0.67 | 0.64 | 0.67 |
|  | [0.58; 3.59] | [0.57; 3.53] | [0.57; 3.57] | [0.57; 3.55] | [0.29; 1.54] | [0.29; 1.54] | [0.28; 1.49] | [0.29; 1.56] |
| Effect of wealthy origin vs poor origin HDI cut at 0.5 | 1.94* | 5.96* |  |  | 1.41 | 1.46 |  |  |
|  | [1.06; 3.56] | [1.65; 21.49] |  |  | [0.93; 2.13] | [0.62; 3.43] |  |  |
| Effect of stays longer than 3 months vs stays shorter than 3 months when women are from poorer countries | 1.96* | 4.72* | 2.94* | 2.78* | 0.79 | 0.81 | 0.57* | 0.65 |
| [1.05; 3.65] | [1.51; 14.72] | [1.35; 6.39] | [1.38; 5.6] | [0.50; 1.25] | [0.38; 1.73] | [0.35; 0.95] | [0.4; 1.05] |
| Effect of stays longer than 3 months vs stays shorter than 3 months when women are from rich countries (HDI cut 0.5) |  | 1.22 |  |  |  | 0.77 |  |  |
|  | [0.59; 2.55] |  |  |  | [0.45; 1.35] |  |  |
| Effect of wealthy origin vs poor origin HDI cut at 0.6 |  |  | 4.71* |  |  |  | 0.28* |  |
|  |  | [1.1; 20.25] |  |  |  | [0.09; 0.94] |  |
| Effect of stays longer than 3 months vs stays shorter than 3 months when women are from rich countries (HDI cut 0.6) |  |  | 0.92 |  |  |  | 2.09 |  |
|  |  | [0.34; 2.48] |  |  |  | [0.77; 5.69] |  |
| Effect of wealthy origin vs poor origin HDI cut at 0.7 |  |  |  | 8.48* |  |  |  | 0.26* |
|  |  |  | [1.78; 40.45] |  |  |  | [0.07; 1.07] |
| Effect of stays longer than 3 months vs stays shorter than 3 months when women are from rich countries (HDI cut 0.7) |  |  |  | 0.38 |  |  |  | 2.09 |
|  |  |  | [0.09; 1.55] |  |  |  | [0.56; 7.86] |
| AIC | 669.98 | 667.57 | 672.28 | 669.45 | 937.37 | 939.36 | 936.38 | 938.14 |
| BIC | 761.57 | 763.52 | 768.23 | 765.40 | 1035.05 | 1041.69 | 1038.71 | 1040.47 |
| Log Likelihood | -313.99 | -311.79 | -314.14 | -312.72 | -447.68 | -447.68 | -446.19 | -447.07 |
| Deviance | 627.98 | 623.57 | 628.28 | 625.45 | 895.37 | 895.36 | 892.38 | 894.14 |
| Num. obs. | 579 | 579 | 579 | 579 | 774 | 774 | 774 | 774 |
| * 1 outside the confidence interval |
| Statistical models |
