# Supplementary material for: Self-Perceived Health among Migrants Seen in Médecins du Monde Free Clinics in Europe: Impact of Length of Stay and Wealth of Country of Origin on Migrants’ Health
Source: Int J Environ Res Public Health. 2019 Dec 4;16(24):4878. doi: 10.3390/ijerph16244878 (PMC6950051; doi:10.3390/ijerph16244878)
Supplement: Supplementary file 1 [file ijerph-16-04878-s001.zip › IJERPH 2019 - 12 - 02/Result Sensitivity Analysis Psy Interactions S8.odt]

Table S8 Sensitivity analysis for Mental SPH, interactions using different cut-off points for Human Development Index. 

|  | Mental Self-Perceived Health |
| --- | --- |
|  | Females | Males |
|  | Model 1 | Model 2 | Model 3 | Model 4 | Model 5 | Model 6 | Model 7 | Model 8 |
|  |  |  |  |  |  |  |  |  |
|  |  |  |  |  |  |  |  |  |
|  | 0.52 | 0.52 | 0.53 | 0.55 | 0.89 | 0.88 | 0.89 | 0.88 |
| 2nd quartile [28;34[ | [0.27; 1.03] | [0.27; 1.03] | [0.27; 1.05] | [0.28; 1.10] | [0.50; 1.56] | [0.50; 1.55] | [0.50; 1.57] | [0.50; 1.56] |
|  | 0.63 | 0.63 | 0.62 | 0.65 | 1.02 | 1.01 | 1.02 | 1.02 |
| 3rd quartile [34;44[ | [0.34; 1.16] | [0.34; 1.16] | [0.34; 1.16] | [0.35; 1.20] | [0.58; 1.77] | [0.58; 1.75] | [0.58; 1.77] | [0.58; 1.77] |
|  | 0.59 | 0.59 | 0.59 | 0.63 | 0.85 | 0.84 | 0.85 | 0.85 |
| 4th quartile [44;85] | [0.32; 1.10] | [0.31; 1.09] | [0.32; 1.11] | [0.34; 1.19] | [0.47; 1.54] | [0.46; 1.53] | [0.47; 1.55] | [0.47; 1.55] |
|  | 4.66 | 4.60 | 5.03 | 5.52 | 2.49 | 2.57 | 2.55 | 2.37 |
| Switzerland | [0.60; 36.39] | [0.59; 35.67] | [0.62; 40.67] | [0.66; 46.51] | [0.54; 11.59] | [0.55; 11.99] | [0.54; 12.00] | [0.49; 11.41] |
|  | 0.77 | 0.78 | 0.84 | 0.98 | 2.74 | 2.89 | 2.81 | 2.55 |
| Spain | [0.22; 2.73] | [0.22; 2.77] | [0.23; 3.03] | [0.26; 3.68] | [0.77; 9.67] | [0.81; 10.36] | [0.77; 10.23] | [0.68; 9.56] |
|  | 0.08* | 0.08* | 0.09* | 0.10* | 0.10* | 0.11* | 0.11* | 0.10* |
| Turkey | [0.02; 0.39] | [0.02; 0.39] | [0.02; 0.42] | [0.02; 0.48] | [0.02; 0.50] | [0.02; 0.51] | [0.02; 0.52] | [0.02; 0.49] |
|  | 0.31 | 0.31 | 0.33 | 0.36 | 0.89 | 0.92 | 0.90 | 0.85 |
| United Kingdom | [0.08; 1.25] | [0.08; 1.24] | [0.08; 1.32] | [0.09; 1.50] | [0.20; 3.95] | [0.21; 4.12] | [0.20; 4.05] | [0.19; 3.90] |
|  | 2.29 | 2.40 | 2.71 | 2.26 | 0.91 | 0.88 | 0.90 | 0.90 |
| Africa | [0.51; 10.27] | [0.53; 10.85] | [0.58; 12.67] | [0.53; 9.66] | [0.36; 2.30] | [0.35; 2.25] | [0.36; 2.21] | [0.37; 2.23] |
|  | 2.76 | 2.92 | 2.43 | 2.43 | 0.47 | 0.45 | 0.48 | 0.52 |
| America | [0.53; 14.31] | [0.56; 15.29] | [0.45; 13.27] | [0.45; 12.97] | [0.14; 1.63] | [0.13; 1.58] | [0.12; 1.88] | [0.14; 1.99] |
|  | 3.98 | 4.34 | 3.81 | 3.62 | 1.73 | 1.72 | 1.77 | 1.75 |
| Asia | [0.85; 18.57] | [0.91; 20.69] | [0.80; 18.13] | [0.80; 16.30] | [0.72; 4.18] | [0.71; 4.16] | [0.71; 4.40] | [0.73; 4.16] |
|  | 2.51 | 2.60 | 2.16 | 1.84 | 0.91 | 0.90 | 0.94 | 1.02 |
| Europe | [0.49; 12.93] | [0.50; 13.50] | [0.40; 11.74] | [0.31; 10.79] | [0.25; 3.33] | [0.25; 3.29] | [0.23; 3.84] | [0.24; 4.30] |
|  | 3.47 | 3.73 | 3.09 | 3.07 | 0.99 | 0.96 | 1.01 | 1.04 |
| Maghreb | [0.61; 19.67] | [0.65; 21.45] | [0.51; 18.61] | [0.55; 17.12] | [0.33; 3.00] | [0.32; 2.92] | [0.29; 3.50] | [0.34; 3.21] |
|  | 0.78 | 0.79 | 0.77 | 0.80 | 0.70 | 0.71 | 0.71 | 0.71 |
| Undocumented | [0.49; 1.24] | [0.49; 1.26] | [0.48; 1.23] | [0.50; 1.28] | [0.46; 1.09] | [0.46; 1.09] | [0.46; 1.09] | [0.46; 1.09] |
|  | 0.75 | 0.76 | 0.76 | 0.78 | 0.54* | 0.54* | 0.54* | 0.54* |
| Asylum Seeker | [0.45; 1.25] | [0.45; 1.27] | [0.46; 1.27] | [0.47; 1.31] | [0.32; 0.91] | [0.32; 0.91] | [0.32; 0.91] | [0.32; 0.90] |
|  | 1.98* | 1.97* | 1.95* | 2.00* | 1.62* | 1.61* | 1.62* | 1.63* |
| Stable Accomodation | [1.25; 3.13] | [1.25; 3.11] | [1.23; 3.09] | [1.26; 3.18] | [1.06; 2.47] | [1.06; 2.46] | [1.06; 2.47] | [1.07; 2.49] |
|  | 1.20 | 1.20 | 1.23 | 1.24 | 0.44* | 0.44* | 0.44* | 0.44* |
| Income satisfaction Enough | [0.67; 2.17] | [0.67; 2.17] | [0.68; 2.22] | [0.69; 2.24] | [0.20; 0.96] | [0.20; 0.95] | [0.20; 0.96] | [0.20; 0.96] |
|  | 1.53 | 1.53 | 1.50 | 1.50 | 2.10* | 2.12* | 2.11* | 2.11* |
| Job | [0.92; 2.54] | [0.92; 2.54] | [0.90; 2.49] | [0.90; 2.50] | [1.30; 3.41] | [1.31; 3.45] | [1.30; 3.42] | [1.30; 3.42] |
|  | 1.78 | 1.77 | 1.82 | 1.83 | 1.63 | 1.63 | 1.63 | 1.63 |
| Not Covered | [0.75; 4.24] | [0.74; 4.23] | [0.76; 4.34] | [0.76; 4.36] | [0.71; 3.74] | [0.71; 3.76] | [0.71; 3.75] | [0.71; 3.75] |
|  | 0.90 | 1.29 |  |  | 1.02 | 1.38 |  |  |
| Effect of stays longer than 3 months vs stays shorter than 3 months when women are from poorer countries | [0.41; 1.97] | [0.36; 4.6] |  |  | [0.55; 1.88] | [0.42; 4.53] |  |  |
| 0.73 | 1.00 | 0.86 | 0.98 | 0.75 | 0.98 | 0.79 | 0.72 |
| Effect of stays longer than 3 months vs stays shorter than 3 months when women are from rich countries (HDI cut 0.5) | [0.39; 1.38] | [0.34; 2.94] | [0.38; 1.93] | [0.48; 2] | [0.41; 1.36] | [0.33; 2.89] | [0.39; 1.61] | [0.38; 1.36] |
| Effect of wealthy origin vs poor origin HDI cut at 0.6 |  | 0.62 |  |  |  | 0.67 |  |  |
|  | [0.29; 1.33] |  |  |  | [0.33; 1.34] |  |  |
| Effect of stays longer than 3 months vs stays shorter than 3 months when women are from rich countries (HDI cut 0.6) |  |  | 1.86 |  |  |  | 1.08 |  |
|  |  | [0.47; 7.4] |  |  |  | [0.3; 3.9] |  |
| Effect of wealthy origin vs poor origin HDI cut at 0.7 |  |  | 0.54 |  |  |  | 0.68 |  |
|  |  | [0.2; 1.45] |  |  |  | [0.26; 1.84] |  |
| Effect of stays longer than 3 months vs stays shorter than 3 months when women are from rich countries (HDI cut 0.7) |  |  |  | 4.11 |  |  |  | 0.74 |
|  |  |  | [0.86; 19.58] |  |  |  | [0.17; 3.31] |
|  |  |  |  | 0.20 |  |  |  | 0.90 |
|  |  |  | [0.05; 0.84] |  |  |  | [0.23; 3.58] |
| AIC | 610.54 | 612.02 | 611.83 | 608.45 | 698.89 | 700.55 | 700.82 | 700.71 |
| BIC | 702.09 | 707.93 | 707.75 | 704.36 | 796.52 | 802.83 | 803.10 | 802.99 |
| Log Likelihood | -284.27 | -284.01 | -283.92 | -282.22 | -328.45 | -328.28 | -328.41 | -328.35 |
| Deviance | 568.54 | 568.02 | 567.83 | 564.45 | 656.89 | 656.55 | 656.82 | 656.71 |
| Num. obs. | 578 | 578 | 578 | 578 | 772 | 772 | 772 | 772 |
| * 1 outside the confidence interval |
| Statistical models |
