# Supplementary material for: Self-Perceived Health among Migrants Seen in Médecins du Monde Free Clinics in Europe: Impact of Length of Stay and Wealth of Country of Origin on Migrants’ Health
Source: Int J Environ Res Public Health. 2019 Dec 4;16(24):4878. doi: 10.3390/ijerph16244878 (PMC6950051; doi:10.3390/ijerph16244878)
Supplement: Supplementary file 1 [file ijerph-16-04878-s001.zip › IJERPH 2019 - 12 - 02/SA - Men - Mental Health S4.odt]

Table S4 Men Mental Health

Sensitivity analysis for mental SPH, for men; SA1 shows results for Length of Stay (LOS) below and above 3 months; SA2 show results for LOS below 3 months, below 10 years and above 3 months, above 10 years; SA3 shows results for LOS below 3 months, below 5 years and above 3 months, above 5 years; Finally, SA4 shows results for LOS below 3 months, below 5 years and above 3 months, above 5 years and below 10 years, above 10 years. 

|  | Mental Health |
| --- | --- |
|  | Men |
|  | SA 1 | SA 2 | SA 3 | SA 4 |
| Age group |  |  |  |  |
| 1st quartile [18;28[ | Ref | Ref | Ref | Ref |
| 2nd quartile [28;34[ | 0.91 [0.52; 1.58] | 0.90 [0.52; 1.58] | 0.96 [0.55; 1.69] | 0.96 [0.55; 1.69] |
| 3rd quartile [34;44[ | 0.98 [0.57; 1.70] | 0.97 [0.55; 1.69] | 1.08 [0.61; 1.92] | 1.06 [0.60; 1.89] |
| 4th quartile [44;85] | 0.80 [0.44; 1.45] | 0.79 [0.43; 1.45] | 0.89 [0.48; 1.64] | 0.86 [0.47; 1.60] |
| Surveyed Countries |  |  |  |  |
| Germany | Ref | Ref | Ref | Ref |
| Switzerland | 3.41 [0.77; 17.04] | 3.47 [0.77; 17.53] | 3.10 [0.69; 15.55] | 3.26 [0.72; 16.47] |
| Spain | 3.15 [0.97; 12.52] | 3.17 [0.98; 12.64] | 3.25 [1.00; 12.91] | 3.39 [1.04; 13.58]* |
| Turkey | 0.12 [0.03; 0.63]* | 0.13 [0.03; 0.65]* | 0.11 [0.02; 0.55]* | 0.11 [0.02; 0.58]* |
| United Kingdom | 1.00 [0.24; 4.76] | 1.01 [0.24; 4.83] | 0.97 [0.23; 4.65] | 1.01 [0.24; 4.86] |
| Region of Origin |  |  |  |  |
| Middle East | Ref | Ref | Ref | Ref |
| Africa | 0.84 [0.35; 2.10] | 0.84 [0.35; 2.09] | 0.82 [0.35; 2.06] | 0.81 [0.34; 2.03] |
| America | 0.44 [0.13; 1.51] | 0.45 [0.13; 1.53] | 0.42 [0.12; 1.45] | 0.43 [0.13; 1.47] |
| Asia | 1.59 [0.70; 3.86] | 1.60 [0.70; 3.88] | 1.51 [0.66; 3.68] | 1.51 [0.65; 3.69] |
| Europe | 0.72 [0.19; 2.62] | 0.73 [0.19; 2.64] | 0.68 [0.18; 2.48] | 0.68 [0.18; 2.49] |
| Maghreb | 0.88 [0.30; 2.64] | 0.87 [0.29; 2.64] | 0.89 [0.30; 2.69] | 0.88 [0.30; 2.66] |
| Housing conditions |  |  |  |  |
| Instable | Ref | Ref | Ref | Ref |
| Stable | 1.61 [1.06; 2.44]* | 1.61 [1.06; 2.45]* | 1.57 [1.04; 2.39]* | 1.58 [1.04; 2.41]* |
| Income satisfaction |  |  |  |  |
| Not Enough | Ref | Ref | Ref | Ref |
| Enough | 0.44 [0.20; 0.92]* | 0.44 [0.20; 0.92]* | 0.47 [0.21; 0.99]* | 0.47 [0.21; 0.98]* |
| Job |  |  |  |  |
| No | Ref | Ref | Ref | Ref |
| Yes | 2.11 [1.32; 3.40]* | 2.11 [1.32; 3.40]* | 2.16 [1.35; 3.49]* | 2.15 [1.34; 3.48]* |
| Insurance coverage |  |  |  |  |
| Yes | Ref | Ref | Ref | Ref |
| No | 1.62 [0.74; 3.81] | 1.61 [0.74; 3.80] | 1.67 [0.76; 3.96] | 1.67 [0.76; 3.97] |
| Immigration Status |  |  |  |  |
| Residence permit | Ref | Ref | Ref | Ref |
| Undocumented | 0.44 [0.24; 0.79]* | 0.44 [0.24; 0.80]* | 0.41 [0.22; 0.75]* | 0.41 [0.23; 0.76]* |
| Asylum Seeker | 0.34 [0.18; 0.63]* | 0.34 [0.18; 0.63]* | 0.32 [0.17; 0.60]* | 0.32 [0.17; 0.61]* |
| Length of Stay |  |  |  |  |
| Sensitivity Analysis 1 |  |  |  |  |
| Length of Stay below 3 months | Ref |  |  |  |
| Length of Stay above 3 months | 0.80 [0.45; 1.45] |  |  |  |
| Sensitivity Analysis 2 |  |  |  |  |
| Length of Stay below 3 months |  | Ref |  |  |
| Length of Stay between 3months and 10 years |  | 0.79 [0.44; 1.44] |  |  |
| Length of Stay above 10 years |  | 0.85 [0.38; 1.93] |  |  |
| Sensitivity Analysis 3 |  |  |  |  |
| Length of Stay below 3 months |  |  | Ref |  |
| Length of Stay between 3 months and 5 years |  |  | 0.88 [0.49; 1.63] |  |
| Length of Stay above 5 years |  |  | 0.63 [0.32; 1.26] |  |
| Sensitivity Analysis 4 |  |  |  |  |
| Length of Stay below 3 months |  |  |  | 1.80 [0.86; 3.76] |
| Length of Stay above three months and Below five years |  |  |  | 1.58 [0.90; 2.81] |
| Length of Stay above 5 years and less than 10 years |  |  |  | Ref |
| Length of Stay Above 10 years |  |  |  | 1.40 [0.68; 2.90] |
| AIC | 692.16 | 694.11 | 692.41 | 693.57 |
| BIC | 785.14 | 791.74 | 790.04 | 795.85 |
| Log Likelihood | -326.08 | -326.05 | -325.21 | -324.79 |
| Deviance | 652.16 | 652.11 | 650.41 | 649.57 |
| Num. obs. | 772 | 772 | 772 | 772 |
