# Supplementary material for: Self-Perceived Health among Migrants Seen in Médecins du Monde Free Clinics in Europe: Impact of Length of Stay and Wealth of Country of Origin on Migrants’ Health
Source: Int J Environ Res Public Health. 2019 Dec 4;16(24):4878. doi: 10.3390/ijerph16244878 (PMC6950051; doi:10.3390/ijerph16244878)
Supplement: Supplementary file 1 [file ijerph-16-04878-s001.zip › IJERPH 2019 - 12 - 02/SA - Men - Physical Health S2.odt]

Table S2 Men Physical Health 

Sensitivity analysis for physical SPH, for men; SA1 shows results for Length of Stay (LOS) below and above 3 months; SA2 show results for LOS below 3 months, below 10 years and above 3 months, above 10 years; SA3 shows results for LOS below 3 months, below 5 years and above 3 months, above 5 years; Finally, SA4 shows results for LOS below 3 months, below 5 years and above 3 months, above 5 years and below 10 years, above 10 years.

|  | Physical Health |
| --- | --- |
|  | Men |
|  | SA 1 | SA 2 | SA 3 | SA 4 |
| Age group |  |  |  |  |
| 1st quartile [18;28[ | Ref | Ref | Ref | Ref |
| 2nd quartile [28;34[ | 0.90 [0.59; 1.37] | 0.91 [0.60; 1.38] | 0.92 [0.60; 1.40] | 0.92 [0.60; 1.40] |
| 3rd quartile [34;44[ | 0.62 [0.40; 0.96]* | 0.63 [0.41; 0.98]* | 0.64 [0.41; 1.00]* | 0.64 [0.41; 1.00] |
| 4th quartile [44;85] | 0.63 [0.37; 1.08] | 0.66 [0.38; 1.13] | 0.65 [0.38; 1.13] | 0.66 [0.38; 1.15] |
| Surveyed Countries |  |  |  |  |
| Germany | Ref | Ref | Ref | Ref |
| Switzerland | 1.17 [0.28; 4.90] | 1.11 [0.26; 4.68] | 1.12 [0.27; 4.71] | 1.09 [0.26; 4.62] |
| Spain | 2.10 [0.74; 6.62] | 2.07 [0.73; 6.54] | 2.14 [0.75; 6.76] | 2.10 [0.73; 6.65] |
| Turkey | 1.63 [0.43; 6.52] | 1.53 [0.40; 6.18] | 1.54 [0.40; 6.24] | 1.50 [0.39; 6.10] |
| United Kingdom | 0.61 [0.16; 2.44] | 0.60 [0.16; 2.38] | 0.61 [0.16; 2.43] | 0.60 [0.16; 2.39] |
| Region of Origin |  |  |  |  |
| Middle East | Ref | Ref | Ref | Ref |
| Africa | 1.21 [0.54; 2.95] | 1.22 [0.54; 2.96] | 1.20 [0.53; 2.92] | 1.21 [0.53; 2.94] |
| America | 0.77 [0.24; 2.49] | 0.74 [0.23; 2.43] | 0.75 [0.23; 2.44] | 0.74 [0.23; 2.41] |
| Asia | 2.22 [0.92; 5.84] | 2.19 [0.91; 5.78] | 2.16 [0.89; 5.73] | 2.17 [0.89; 5.73] |
| Europe | 1.80 [0.51; 6.46] | 1.78 [0.50; 6.36] | 1.77 [0.50; 6.33] | 1.76 [0.49; 6.31] |
| Maghreb | 1.91 [0.67; 5.65] | 1.91 [0.67; 5.65] | 1.92 [0.68; 5.68] | 1.91 [0.67; 5.67] |
| Housing conditions |  |  |  |  |
| Instable | Ref | Ref | Ref | Ref |
| Stable | 1.30 [0.92; 1.83] | 1.29 [0.91; 1.82] | 1.29 [0.91; 1.82] | 1.29 [0.91; 1.82] |
| Income satisfaction |  |  |  |  |
| Not Enough | Ref | Ref | Ref | Ref |
| Enough | 0.95 [0.45; 1.96] | 0.97 [0.45; 2.00] | 0.98 [0.46; 2.04] | 0.98 [0.46; 2.04] |
| Job |  |  |  |  |
| No | Ref | Ref | Ref | Ref |
| Yes | 1.81 [1.26; 2.61]* | 1.82 [1.27; 2.63]* | 1.82 [1.26; 2.62]* | 1.82 [1.27; 2.63]* |
| Insurance coverage |  |  |  |  |
| Yes | Ref | Ref | Ref | Ref |
| No | 0.64 [0.29; 1.51] | 0.65 [0.29; 1.52] | 0.65 [0.29; 1.53] | 0.65 [0.29; 1.53] |
| Immigration Status |  |  |  |  |
| Residence permit | Ref | Ref | Ref | Ref |
| Undocumented | 1.11 [0.69; 1.82] | 1.09 [0.67; 1.78] | 1.09 [0.67; 1.78] | 1.08 [0.66; 1.77] |
| Asylum Seeker | 0.60 [0.35; 1.02] | 0.59 [0.35; 1.01] | 0.59 [0.35; 1.01] | 0.59 [0.34; 1.00] |
| Length of Stay |  |  |  |  |
| Sensitivity Analysis 1 |  |  |  |  |
| Length of Stay below 3 months | Ref |  |  |  |
| Length of Stay above 3 months | 0.72 [0.46; 1.14] |  |  |  |
| Sensitivity Analysis 2 |  |  |  |  |
| Length of Stay below 3 months |  | Ref |  |  |
| Length of Stay between 3months and 10 years |  | 0.74 [0.47; 1.16] |  |  |
| Length of Stay above 10 years |  | 0.57 [0.25; 1.23] |  |  |
| Sensitivity Analysis 3 |  |  |  |  |
| Length of Stay below 3 months |  |  | Ref |  |
| Length of Stay between 3 months and 5 years |  |  | 0.75 [0.47; 1.19] |  |
| Length of Stay above 5 years |  |  | 0.63 [0.35; 1.16] |  |
| Sensitivity Analysis 4 |  |  |  |  |
| Length of Stay below 3 months |  |  |  | 1.47 [0.77; 2.86] |
| Length of Stay above three months and Below five years |  |  |  | 1.10 [0.63; 1.95] |
| Length of Stay above 5 years and less than 10 years |  |  |  | Ref |
| Length of Stay Above 10 years |  |  |  | 0.82 [0.36; 1.77] |
| AIC | 938.23 | 939.68 | 939.83 | 941.57 |
| BIC | 1031.26 | 1037.36 | 1037.51 | 1043.90 |
| Log Likelihood | -449.11 | -448.84 | -448.91 | -448.78 |
| Deviance | 898.23 | 897.68 | 897.83 | 897.57 |
| Num. obs. | 774 | 774 | 774 | 774 |
