# Supplementary material for: Self-Perceived Health among Migrants Seen in Médecins du Monde Free Clinics in Europe: Impact of Length of Stay and Wealth of Country of Origin on Migrants’ Health
Source: Int J Environ Res Public Health. 2019 Dec 4;16(24):4878. doi: 10.3390/ijerph16244878 (PMC6950051; doi:10.3390/ijerph16244878)
Supplement: Supplementary file 1 [file ijerph-16-04878-s001.zip › IJERPH 2019 - 12 - 02/SA - Women - Mental Health S3.odt]

Table S3 Women Mental Health

Sensitivity analysis for mental SPH, for women; SA1 shows results for Length of Stay (LOS) below and above 3 months; SA2 show results for LOS below 3 months, below 10 years and above 3 months, above 10 years; SA3 shows results for LOS below 3 months, below 5 years and above 3 months, above 5 years; Finally, SA4 shows results for LOS below 3 months, below 5 years and above 3 months, above 5 years and below 10 years, above 10 years.

|  | Mental Health |
| --- | --- |
|  | Women |
|  | SA 1 | SA 2 | SA 3 | SA 4 |
| Age group |  |  |  |  |
| 1st quartile [18;28[ | Ref | Ref | Ref | Ref |
| 2nd quartile [28;34[ | 0.52 [0.27; 1.00] | 0.51 [0.26; 0.99]* | 0.52 [0.26; 1.00] | 0.52 [0.26; 1.01] |
| 3rd quartile [34;44[ | 0.63 [0.34; 1.15] | 0.64 [0.35; 1.16] | 0.63 [0.34; 1.15] | 0.63 [0.34; 1.15] |
| 4th quartile [44;85] | 0.59 [0.32; 1.09] | 0.62 [0.33; 1.15] | 0.59 [0.31; 1.10] | 0.60 [0.32; 1.13] |
| Surveyed Countries |  |  |  |  |
| Germany | Ref | Ref | Ref | Ref |
| Switzerland | 4.85 [0.68; 45.03] | 4.88 [0.68; 45.35] | 4.84 [0.67; 44.95] | 4.87 [0.68; 45.29] |
| Spain | 0.74 [0.21; 2.61] | 0.73 [0.21; 2.57] | 0.74 [0.21; 2.62] | 0.72 [0.20; 2.56] |
| Turkey | 0.08 [0.02; 0.38]* | 0.08 [0.02; 0.38]* | 0.08 [0.02; 0.38]* | 0.08 [0.02; 0.37]* |
| United Kingdom | 0.31 [0.08; 1.19] | 0.31 [0.08; 1.21] | 0.30 [0.08; 1.20] | 0.30 [0.07; 1.19] |
| Region of Origin |  |  |  |  |
| Middle East | Ref | Ref | Ref | Ref |
| Africa | 2.22 [0.59; 11.38] | 2.24 [0.60; 11.51] | 2.20 [0.59; 11.35] | 2.20 [0.59; 11.31] |
| America | 2.49 [0.55; 14.41] | 2.51 [0.55; 14.57] | 2.48 [0.55; 14.38] | 2.55 [0.56; 14.78] |
| Asia | 3.57 [0.90; 19.05] | 3.55 [0.90; 18.92] | 3.55 [0.89; 18.99] | 3.49 [0.88; 18.67] |
| Europe | 2.32 [0.51; 13.27] | 2.37 [0.52; 13.52] | 2.31 [0.50; 13.24] | 2.34 [0.51; 13.36] |
| Maghreb | 3.39 [0.67; 21.43] | 3.59 [0.70; 22.73] | 3.38 [0.66; 21.36] | 3.50 [0.68; 22.20] |
| Housing conditions |  |  |  |  |
| Instable | Ref | Ref | Ref | Ref |
| Stable | 1.96 [1.26; 3.08]* | 1.96 [1.26; 3.09]* | 1.96 [1.26; 3.08]* | 1.95 [1.25; 3.07]* |
| Income satisfaction |  |  |  |  |
| Not Enough | Ref | Ref | Ref | Ref |
| Enough | 1.22 [0.69; 2.17] | 1.21 [0.68; 2.15] | 1.22 [0.69; 2.17] | 1.20 [0.67; 2.14] |
| Job |  |  |  |  |
| No | Ref | Ref | Ref | Ref |
| Yes | 1.47 [0.90; 2.40] | 1.49 [0.91; 2.43] | 1.46 [0.89; 2.41] | 1.46 [0.89; 2.40] |
| Insurance coverage |  |  |  |  |
| Yes | Ref | Ref | Ref | Ref |
| No | 1.79 [0.80; 4.46] | 1.79 [0.80; 4.47] | 1.80 [0.79; 4.52] | 1.84 [0.81; 4.63] |
| Immigration Status |  |  |  |  |
| Residence permit | Ref | Ref | Ref | Ref |
| Undocumented | 0.91 [0.50; 1.68] | 0.90 [0.49; 1.67] | 0.92 [0.50; 1.71] | 0.91 [0.49; 1.69] |
| Asylum Seeker | 0.73 [0.39; 1.40] | 0.72 [0.38; 1.38] | 0.73 [0.39; 1.41] | 0.71 [0.37; 1.37] |
| Length of Stay |  |  |  |  |
| Sensitivity Analysis 1 |  |  |  |  |
| Length of Stay below 3 months | Ref |  |  |  |
| Length of Stay above 3 months | 0.70 [0.38; 1.33] |  |  |  |
| Sensitivity Analysis 2 |  |  |  |  |
| Length of Stay below 3 months |  | Ref |  |  |
| Length of Stay between 3months and 10 years |  | 0.74 [0.40; 1.40] |  |  |
| Length of Stay above 10 years |  | 0.56 [0.22; 1.37] |  |  |
| Sensitivity Analysis 3 |  |  |  |  |
| Length of Stay below 3 months |  |  | Ref |  |
| Length of Stay between 3 months and 5 years |  |  | 0.70 [0.37; 1.34] |  |
| Length of Stay above 5 years |  |  | 0.72 [0.35; 1.49] |  |
| Sensitivity Analysis 4 |  |  |  |  |
| Length of Stay below 3 months |  |  |  | 1.26 [0.59; 2.68] |
| Length of Stay above three months and Below five years |  |  |  | 0.89 [0.52; 1.53] |
| Length of Stay above 5 years and less than 10 years |  |  |  | Ref |
| Length of Stay Above 10 years |  |  |  | 0.71 [0.31; 1.53] |
| AIC | 609.68 | 611.75 | 611.67 | 612.90 |
| BIC | 696.87 | 703.34 | 703.22 | 708.81 |
| Log Likelihood | -284.84 | -284.87 | -284.83 | -284.45 |
| Deviance | 569.68 | 569.75 | 569.67 | 568.90 |
| Num. obs. | 578 | 579 | 578 | 578 |
