# Supplementary material for: Self-Perceived Health among Migrants Seen in Médecins du Monde Free Clinics in Europe: Impact of Length of Stay and Wealth of Country of Origin on Migrants’ Health
Source: Int J Environ Res Public Health. 2019 Dec 4;16(24):4878. doi: 10.3390/ijerph16244878 (PMC6950051; doi:10.3390/ijerph16244878)
Supplement: Supplementary file 1 [file ijerph-16-04878-s001.zip › IJERPH 2019 - 12 - 02/SA - Women - Physical Health S1.odt]

Table S1 Women Physical Health 

Sensitivity analysis for physical SPH, for women; SA1 shows results for Length of Stay (LOS) below and above 3 months; SA2 show results for LOS below 3 months, below 10 years and above 3 months, above 10 years; SA3 shows results for LOS below 3 months, below 5 years and above 3 months, above 5 years; Finally, SA4 shows results for LOS below 3 months, below 5 years and above 3 months, above 5 years and below 10 years, above 10 years.

| Physical Health |
| --- |
| Women |
|  | SA 1 | SA 2 | SA 3 | SA 4 |
| Age group |  |  |  |  |
| 1st quartile [18;28[ | Ref | Ref | Ref | Ref |
| 2nd quartile [28;34[ | 1.08 [0.62; 1.91] | 1.08 [0.62; 1.91] | 1.07 [0.61; 1.90] | 1.08 [0.61; 1.91] |
| 3rd quartile [34;44[ | 0.66 [0.37; 1.18] | 0.66 [0.37; 1.19] | 0.65 [0.36; 1.17] | 0.65 [0.36; 1.17] |
| 4th quartile [44;85] | 0.67 [0.36; 1.24] | 0.71 [0.38; 1.33] | 0.66 [0.35; 1.23] | 0.69 [0.37; 1.29] |
| Surveyed Countries |  |  |  |  |
| Germany | Ref | Ref | Ref | Ref |
| Switzerland | 6.32 [0.81; 51.76] | 6.41 [0.82; 52.89] | 6.27 [0.81; 51.34] | 6.35 [0.82; 52.22] |
| Spain | 0.55 [0.15; 2.14] | 0.55 [0.15; 2.14] | 0.53 [0.14; 2.12] | 0.52 [0.13; 2.06] |
| Turkey | 0.93 [0.20; 4.50] | 0.93 [0.20; 4.53] | 0.91 [0.19; 4.45] | 0.90 [0.19; 4.41] |
| United Kingdom | 0.39 [0.09; 1.70] | 0.40 [0.09; 1.76] | 0.38 [0.09; 1.68] | 0.38 [0.09; 1.67] |
| Region of Origin |  |  |  |  |
| Middle East | Ref | Ref | Ref | Ref |
| Africa | 2.89 [0.86; 13.48] | 2.94 [0.88; 13.72] | 2.87 [0.85; 13.37] | 2.86 [0.85; 13.35] |
| America | 9.17 [2.12; 50.68]* | 9.41 [2.17; 52.17]* | 9.15 [2.12; 50.56]* | 9.58 [2.21; 53.13]* |
| Asia | 3.78 [0.97; 19.33] | 3.78 [0.97; 19.34] | 3.73 [0.96; 19.14] | 3.66 [0.94; 18.79] |
| Europe | 2.77 [0.58; 16.00] | 2.85 [0.60; 16.49] | 2.75 [0.58; 15.91] | 2.80 [0.59; 16.22] |
| Maghreb | 4.19 [0.85; 25.14] | 4.50 [0.91; 27.17] | 4.14 [0.84; 24.91] | 4.35 [0.88; 26.30] |
| Housing conditions |  |  |  |  |
| Instable | Ref | Ref | Ref | Ref |
| Stable | 1.79 [1.19; 2.71]* | 1.79 [1.19; 2.71]* | 1.78 [1.18; 2.70]* | 1.77 [1.17; 2.68]* |
| Income satisfaction |  |  |  |  |
| Not Enough | Ref | Ref | Ref | Ref |
| Enough | 1.39 [0.76; 2.54] | 1.38 [0.75; 2.52] | 1.39 [0.76; 2.54] | 1.36 [0.74; 2.49] |
| Job |  |  |  |  |
| No | Ref | Ref | Ref | Ref |
| Yes | 1.33 [0.85; 2.07] | 1.33 [0.85; 2.09] | 1.32 [0.84; 2.06] | 1.31 [0.83; 2.05] |
| Insurance coverage |  |  |  |  |
| Yes | Ref | Ref | Ref | Ref |
| No | 1.36 [0.58; 3.58] | 1.36 [0.58; 3.59] | 1.38 [0.59; 3.66] | 1.43 [0.60; 3.78] |
| Immigration Status |  |  |  |  |
| Residence permit | Ref | Ref | Ref | Ref |
| Undocumented | 0.69 [0.38; 1.25] | 0.68 [0.38; 1.23] | 0.70 [0.39; 1.28] | 0.69 [0.38; 1.25] |
| Asylum Seeker | 0.50 [0.27; 0.93]* | 0.48 [0.26; 0.91]* | 0.50 [0.27; 0.94]* | 0.48 [0.25; 0.90]* |
| Length of Stay |  |  |  |  |
| Sensitivity Analysis 1 |  |  |  |  |
| Length of Stay below 3 months | Ref |  |  |  |
| Length of Stay above 3 months | 2.13 [1.17; 4.03]* |  |  |  |
| Sensitivity Analysis 2 |  |  |  |  |
| Length of Stay below 3 months |  | Ref |  |  |
| Length of Stay between 3months and 10 years |  | 2.24 [1.23; 4.26]* |  |  |
| Length of Stay above 10 years |  | 1.54 [0.59; 3.94] |  |  |
| Sensitivity Analysis 3 |  |  |  |  |
| Length of Stay below 3 months |  |  | Ref |  |
| Length of Stay between 3 months and 5 years |  |  | 2.10 [1.14; 4.01]* |  |
| Length of Stay above 5 years |  |  | 2.25 [1.08; 4.85]* |  |
| Sensitivity Analysis 4 |  |  |  |  |
| Length of Stay below 3 months |  |  |  | 0.38 [0.17; 0.83]* |
| Length of Stay above three months and Below five years |  |  |  | 0.82 [0.47; 1.43] |
| Length of Stay above 5 years and less than 10 years |  |  |  | Ref |
| Length of Stay Above 10 years |  |  |  | 0.61 [0.26; 1.38] |
| AIC | 671.68 | 673.51 | 673.61 | 674.23 |
| BIC | 758.91 | 765.13 | 765.20 | 770.18 |
| Log Likelihood | -315.84 | -315.75 | -315.81 | -315.11 |
| Deviance | 631.68 | 631.51 | 631.61 | 630.23 |
| Num. obs. | 579 | 580 | 579 | 579 |
